# Paternal Incarceration and Adolescent Delinquency: Role of Father Engagement and Early Child Behavior Problems

**DOI:** 10.1007/s11121-024-01734-2

**Published:** 2024-10-02

**Authors:** Abigail J. Anderson, Christopher C. Henrich, Sylvie Mrug

**Affiliations:** University of Alabama at Birmingham, Birmingham, USA

**Keywords:** Paternal incarceration, Delinquency, Mediation, Father engagement, Child problem behaviors

## Abstract

Paternal incarceration is an important predictor of teen delinquency, but the factors that may explain this relationship—such as early child problem behaviors and level of father engagement—have not been adequately explored. The current longitudinal study examined paternal history of incarceration as a predictor of teen self-reported delinquency over a 15-year gap, considering early child problem behaviors and father engagement as mediators. Sex differences in these relationships were also evaluated. This four-wave longitudinal study included an analytic sample of 4897 teens who participated in the birth-cohort Future of Families and Child Well-Being Study. Mothers and fathers were interviewed shortly after the focal child’s birth and were then reassessed in follow-up interviews at child ages 1, 3, 5, 9, and 15. The focal children were interviewed at ages 9 and 15. Results showed that paternal prior incarceration at year 1 was associated with greater child behavior problems and father engagement at year 5; however, those relationships disappeared by age 9. Paternal history of incarceration was not related to teen delinquency, but child behavior problems at age 9 were directly related to subsequent engagement in delinquent behaviors. Paternal current incarceration was related to subsequent father engagement but was not associated with later child behaviors. No significant indirect pathways emerged, indicating a lack of support for mediation. No sex differences in these relationships were observed. Overall, the findings underscore the complexity of the relationships between paternal incarceration, child behavior, and father engagement in the emergence of delinquent behaviors.

**Supplementary Information:**

The online version contains supplementary material available at 10.1007/s11121-024-01734-2.

Juvenile delinquency has been linked to adverse long-term consequences for youth, their families, and the surrounding community, including problems with educational attainment, employment, substance abuse, future criminal behaviors, and a financial burden on the criminal justice system (Basto-Pereira et al., 2015; Carter, 2019). One important risk factor for teen delinquency is parental incarceration (Porter & King, 2015), which can have a lasting effect on children and adolescents (Poehlmann-Tynan & Turney, 2021; Uggen & McElrath, 2014). Fathers account for approximately 93% of incarcerated parents (Maruschak et al., 2021) and yet are typically underrepresented in literature on child outcomes (Davison et al., 2017; O’Hara & Fisher, 2010). Despite this, research has indicated that rates of delinquency are higher for teens with a father who has a history of incarceration (Roettger & Swisher, 2011), with one study finding that paternal incarceration prior to the child’s birth was related to children’s delinquency at age 19 (Murray et al., 2007).

However, the impact of incarceration prior to early childhood is understudied. In particular, it is not known whether a history of paternal incarceration has differential effects on children’s development than concurrent incarceration throughout childhood. Concurrent paternal incarceration is associated with a myriad of co-occurring changes for children, with direct separation, and therefore lower levels of engagement, from their father being at the forefront. Concurrent incarceration is also related to numerous changes to family structure including relationship disruptions, strained resources, and heightened caregiver stress (Schwartz-Soicher et al., 2011; Turney, 2015). In comparison, paternal incarceration history is associated with increased difficulties in securing jobs (Pettit & Lyons, 2009) and retaining job-related benefits (Turney, 2017b). Father’s ability to contribute financially is also reduced well after release (Geller et al., 2011). With this, children who have a father with a history of incarceration may have greater exposure to other early life adversities due to the repercussions of incarceration on the fathers themselves, such as limited resources (Sykes & Pettit, 2015), which may begin an adverse trajectory that can have a long-term impact on children, even if the incarceration occurs prior to early childhood.

The relationship between paternal incarceration and the development of juvenile delinquency may be explained through multiple mediating variables such as environmental factors, genetics, and behavioral imitation (Besemer et al., 2017; Farrington, 2011). In this study, we focused on two putative mediators of the relationship between paternal incarceration and teen delinquency: child behavior problems and father engagement. Because early child behavior problems are considered an important risk factor for later delinquency (Assink et al., 2015; Thompson et al., 2011), the robust effect of paternal incarceration on children’s behavior problems (Turney, 2017a; Wildeman, 2010) may lead to delinquency during the teen years. Additionally, children’s behaviors in response to paternal incarceration may be shaped by disruptions occurring within their families, such as lower father engagement. Father engagement is often seen as a crucial part of a child’s immediate environment and plays a significant role in shaping their development (Lamb, 2010). Developmental contextualism posits that children’s development is influenced by multiple layers of context including individual factors, such as behavior problems, and family factors, such as engagement (Lerner & Kauffman, 1985). From this perspective, child development is the result of reciprocal relationships between the individual and their environment over time. Paternal incarceration could disrupt the family context which could lead to changes in father engagement as well as the emergence of early behavior problems. These factors may then evoke differences in each other over the transition to adolescence, leading to a heightened risk for delinquency. Taken together, understanding the intervening roles that child behavior problems and father engagement play in the development of delinquency can provide important information about target areas for teen delinquency prevention in families with fathers who have a history of incarceration.

Father engagement is a complex construct that often includes different conceptualizations across the literature (Lamb, 2000). However, in general, paternal incarceration has been shown to be associated with lower levels of father engagement (Dwyer Emory, 2018; Geller, 2013; Swisher & Waller, 2008; Woldoff & Washington, 2008). Further, father engagement has been shown to be positively related to the social, behavioral, and psychological outcomes of children (Sarkadi et al., 2008), whereas low levels of father engagement are a contributing factor to negative child outcomes such as problematic behaviors (Tautolo et al., 2015). Furthermore, a previous study found that child-reported attachment to fathers mediated the association between paternal incarceration and engagement in delinquency (Porter & King, 2015).

Additionally, incarceration of a parent is related to higher externalizing behavior problems in early childhood (Geller et al., 2009; Wilbur et al., 2007), which typically emerge as increased aggression, attention problems, and delinquency (Murray & Farrington, 2008; Roettger & Swisher, 2011). In turn, early emergence of child problem behaviors has long been identified as a risk factor for teen delinquency (Assink et al., 2015; Thompson et al., 2011). If left untreated, early problem behaviors can lead to severe long-term consequences and maladjustment (Gargano et al., 2018; Thompson et al., 2011).

However, the relationship between child problem behaviors and father engagement is complex and often bidirectional. It may be that parents mold their children’s behaviors, and that, in turn, children also play a part in shaping their parents (Burke et al., 2008; Leve & Cicchetti, 2016) . Higher levels of father engagement are associated with fewer externalizing behaviors in their children (Dwyer Emory, 2018; King & Sobolewski, 2006), and higher quality father-child relationships are linked to lower rates of delinquency (Hoeve et al., 2009). Father’s engagement has been found to be protective against children and adolescents’ participation in risky and delinquent behaviors (Goncy & van Dulmen, 2010; Yoder et al., 2016). Conversely, another study could not establish that father engagement consistently predicted lower levels of child problem behaviors across different age groups over the course of early to middle childhood (Flouri et al., 2016). Therefore, clarification of the potential bidirectional relationship between child behavior problems and father engagement needs more longitudinal investigation.

Another key question when considering the relationships between paternal incarceration, early child behavior problems, father engagement, and teen delinquency is whether these associations vary by child sex. Sex differences in delinquency are well-documented, with being male representing one of the most salient predictors of criminal behavior (Bright et al., 2017; Steffensmeier & Schwartz, 2009). Preschool-aged boys are more likely to experience externalizing behavior problems than girls (Mayes et al., 2020; Paz et al., 2021). Fathers are typically less engaged with female children (Lundberg et al., 2007; Ogg & Anthony, 2019), so paternal incarceration may be more detrimental to sons. Research on sex differences in the relation between paternal incarceration and teen delinquency has been mixed. Some studies have suggested differential effects of paternal incarceration on behavior problems for sons and daughters (Geller et al., 2009; Wildeman, 2010), with there being a stronger association for boys (Foster & Hagan, 2013). Having a father who was incarcerated also increases boys’ risk for delinquent behaviors (Murray & Farrington, 2008). However, a few studies did not find sex differences in the relationship between paternal incarceration and behavior problems in their children and teens (Murray & Farrington, 2005; Sampson & Loeffler, 2010).

We sought to advance our understanding of the associations between paternal history of incarceration prior to child age 1, paternal current incarceration at ages 5 and 9, child behavior problems at ages 5 and 9, and level of father engagement at ages 5 and 9 in predicting teen delinquency using a large, longitudinal data set from the Future of Families and Child Wellbeing Study (FFCWS). Previous research on paternal incarceration using this cohort has typically evidenced the adverse effects of recent or concurrent incarceration on child outcomes in early childhood and adolescence (e.g., Dwyer Emory, 2018; Turney, 2017a). Above and beyond the findings in those studies, we considered both paternal incarceration that occurs before child age 1 and incarceration during childhood and the relationships with teen delinquency over a 15-year period to consider the differential impacts of incarceration timing. Moreover, we examined whether the association between paternal history of incarceration and child delinquency is mediated by early child behavior problems and level of father engagement. We also evaluated sex differences in these relationships.

Consistent with prior research, we expected both paternal history of incarceration prior to early childhood and incarceration during childhood to be associated with higher teen delinquency. In addition, we hypothesized that the association between paternal history of incarceration and later teen delinquency would be partially mediated by greater early problem behaviors and lower father involvement. Lastly, we expected that the relationships of incarceration with higher behavior problems, lower father engagement, and higher delinquency would be stronger for boys.

## Method

### Participants and Procedures

We conducted a secondary analysis of data from the Future of Families and Child Well-Being Study (FFCWS). FFCWS is a longitudinal, birth-cohort study that followed nearly 5000 children from 20 large, urban US cities beginning in 1998. Mothers and fathers were interviewed shortly after the focal child’s birth and then were reassessed in follow-up interviews at child ages 1, 3, 5, 9, and 15. Interviews were first conducted with the focal children at age 9, with reassessment occurring at age 15. Data from child ages 1, 5, 9, and 15 were utilized in the current project. The analytic sample (*N* = 4897) included children from the age 15 survey for whom information on paternal incarceration prior to child age 1 was available.

### Measures

#### Paternal Incarceration

##### History of Incarceration

At the age 1 assessment, mothers and fathers were asked whether fathers had ever been incarcerated and if the father was currently incarcerated. Fathers who had a history of incarceration prior to the age 1 assessment or were currently incarcerated during that time were coded 1, while fathers without a history of incarceration were coded 0.

##### Current Incarceration

At ages 5 and 9, mothers and fathers were asked whether the father was currently incarcerated. Fathers who were currently incarcerated were coded 1, while fathers who were not incarcerated were coded 0.

#### Early Child Behavior Problems

At ages 5 and 9, primary caregivers, most commonly mothers, reported on the child’s externalizing behavior problems using the Child Behavior Checklist (Achenbach & Rescorla, 2000). The 30-item Aggression and Delinquent subscales assessed behaviors such as tantrums, sudden changes in mood or feelings, threatening people, being unusually loud, lying or cheating, vandalizing, and setting fires. Higher scores indicate more problem behaviors. Internal consistency was good in the current study (*α* = 0.87–0.90).

#### Father Engagement

During the age 5 assessment, the primary caregiver reported on father engagement with 8 items probing the frequency per week that fathers engaged in certain behaviors with their children, such as playing games like “peek-a-boo,” singing songs, reading stories, and hugging or showing affection. In the current study, this scale demonstrated strong internal consistency (*α* = 0.89). At child age 9, the primary caregiver provided information on father engagement. The Father Involvement scale used 10 items to measure how often in the past month the father and child did certain activities together, such as household chores, outdoor activities, and homework. Items were measured on a 4-point Likert scale ranging from 0 (never) to 4 (every day). The Father Involvement Scale demonstrated strong internal consistency (*α* = 0.92).

#### Teen Delinquency

At age 15, teen self-reported delinquency was measured. Questions about delinquent behaviors in FFCWS were adopted from similar measures in the National Longitudinal Study of Adolescent Health. Teens self-reported whether they engaged in 13 different delinquent behaviors in the past year (0 = No, 1 = Yes), such as “gotten into a serious physical fight,” “gone into a house or building to steal something,” and “driven a car without it’s owner’s permission.” Items were summed with higher scores indicating more delinquent behaviors. Teen-reported delinquency demonstrated acceptable internal consistency (*α* = 0.74).

#### Covariates

At child age 1, father’s race (dummy variables for less than high school, some college, or college degree, with high school diploma as the reference group), education (dummy variables for African American, Hispanic, and other race with White as the reference group), and family income-to-poverty ratio (family household income divided by the poverty threshold) were measured, as well as mother-reported child temperament using six items from the Child’s Emotionality and Shyness Scale (Buss & Plomin, 1984). Mothers reported whether the child often fusses and cries, gets upset easily, or tends to be shy. Questions were measured on a 5-point scale (1 = not at all like my child to 5 = very much like my child). Questions were reverse coded as necessary with higher scores indicating a more difficult temperament. The scale evidenced poor internal consistency (*α* = 0.51), which is consistent with reliabilities from other studies using and validating the measure (e.g., Mathiesen & Tambs, 1999).

### Data Analytic Plan

Preliminary analyses were calculated using Mplus v.8.8 including means, standard deviations, and bivariate correlations among all variables. We examined missing data to identify whether demographic variables differed between those retained by age 15 versus those not retained using SPSS version 29. We conducted a Confirmatory Factor Analysis (CFA) in Mplus using the ML estimator to model the latent factors for child problem behaviors and father’s engagement at child ages 5 and 9. Items on the two scales were parceled with random assignment and without replacement, as recommended for questionaries with numerous items (Little et al., 2002). The 30 items from the Child Behavior checklist were averaged together to create six parcels. For father engagement, at child age 5, the eight items were averaged to create four parcels, with the 10-item Father Involvement Scale averaged to create five parcels at child age 9.

For the main analysis, we used structural equation modeling to examine the potential mediating roles of father engagement and early child behavior problems in the relationship between paternal history of incarceration prior to child age 1, as well as current incarceration at years 5 and 9, and teen self-reported delinquency at age 15, using Mplus with full information maximum likelihood (FIML) estimation which preserves the full sample size by using all available data and minimizes bias when data are missing at random (Wothke, 2000). Full information maximum likelihood allowed for an analytic sample of 4897 participants to be utilized. Model fit was evaluated with comparative fit index (CFI) and root mean square error of approximation (RMSEA), with CFIs greater than 0.95 and RMSEA less than 0.06 indicating good model fit (Hu & Bentler, 1999).

**Fig. 1:**
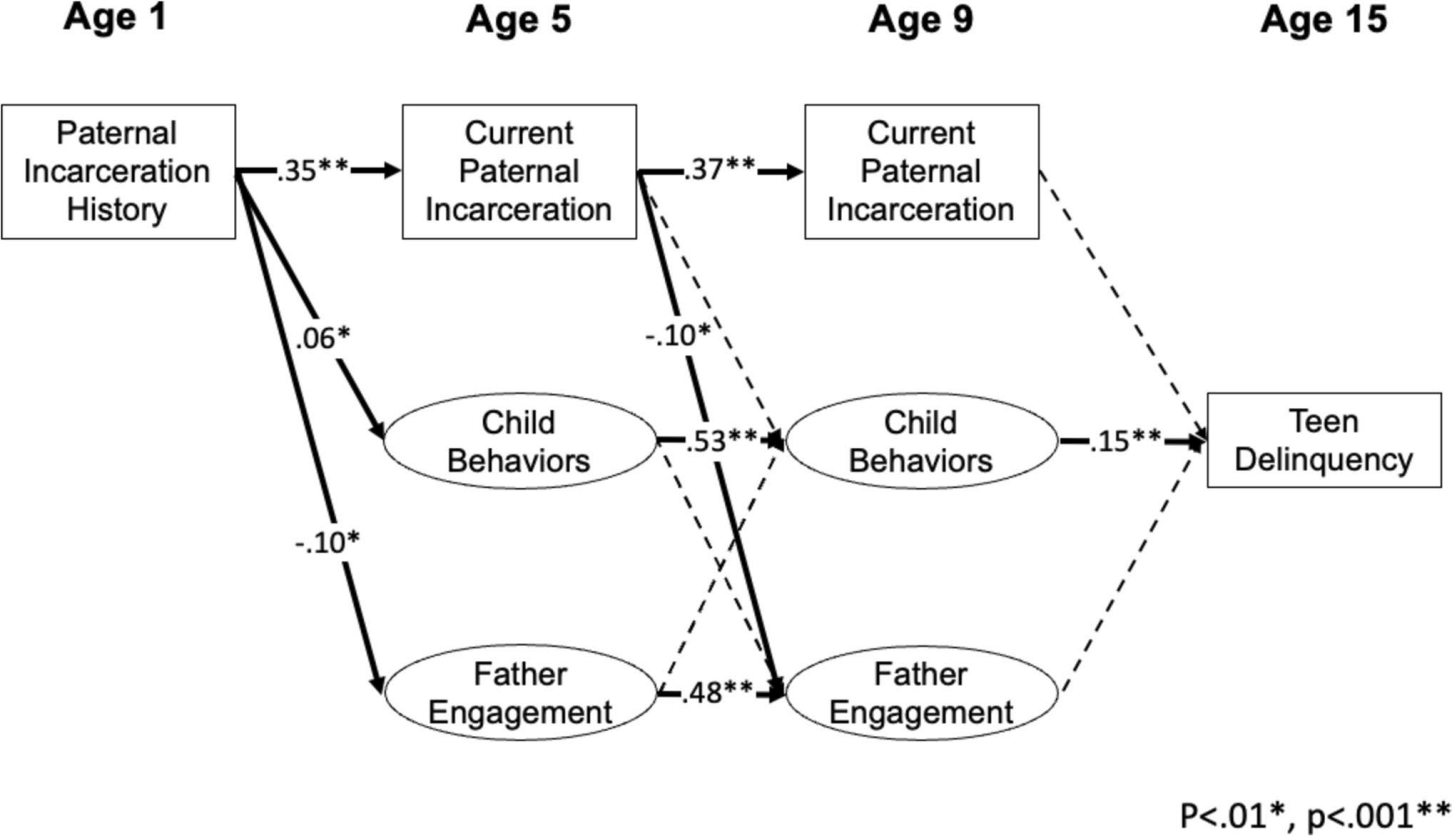
Structural equation model with standardized coefficients. Mediation model. Standardized path coefficients are shown. Solid paths indicate a significant effect; dashed paths are nonsignificant

As shown in Fig. 1, paternal history of incarceration at child-age 1 was expected to be associated with child behavior problems, father engagement, and current incarceration at age 5, child behavior problems and father engagement at age 9, and teen delinquency at age 15. Child behavior problems, father engagement, and current incarceration at age 5 were also expected to be associated with both child behavior problems and father engagement at age 9, as well as teen delinquency at age 15. Lastly, it was expected that child behavior problems, father engagement, and current incarceration at age 9 would predict teen delinquency at age 15. All paths were adjusted for the covariates of paternal education, paternal race, child temperament, and family income-to-poverty ratio.

Indirect effects were tested using bias-corrected bootstrapping with 1000 bootstrap samples. Sex differences in all paths were tested with multigroup structural equation models. Equality constraints were imposed on all paths across sex, and the fit of this constrained model was compared to the fit of an unconstrained model where all parameters were allowed to vary by sex using the chi-square difference test. Better fit of the unconstrained model indicated the presence of sex differences and was followed by comparing the fully constrained model to models with one path freed at a time to identify paths that varied by sex.

## Results

### Preliminary Analyses

Descriptive statistics for all measures used in the analyses are reported in Table 1. Bivariate correlations among the main study variables are reported in Table 2. Paternal incarceration was positively correlated with child behavior problems at both ages 5 and 9, and teen delinquency, and negatively correlated with level of father engagement at ages 5 and 9. Early child behavior problems at age 5 were positively correlated with subsequent child behavior problems and delinquent behaviors and negatively related to father engagement at both ages 5 and 9. Father engagement at age 9 was negatively correlated with teen delinquency.

**Table 1:** Descriptive for study variables

	*N*	Mean	*SD*	Min	Max	%
Paternal incarceration history (age 1)	4388			0	1	8.57%
Child sex (female)	4897			1	2	47.80%
Paternal education (age 1)	3344					
Less than high school				0	1	30.68%
Some college				0	1	25.27%
College				0	1	12.53%
Poverty ratio (age 1)	3365	2.53	3.12	0	57.5	
Child temperament (age 1)	4298	15.55	4.58	6	30	
Paternal race/ethnicity (age 1)	4871					
Black				0	1	49.41%
Hispanic				0	1	27.80%
Other				0	1	4.43%
Child behavior problems (age 5)		0	.19			
Father engagement (age 5)		0	1.66			
Father incarceration (age 5)	3985			0	1	8.13%
Child behavior problems (age 9)		0	.18			
Father engagement (age 9)		0	.97			
Father incarceration (age 9)	3735			0	1	6.53%
Teen delinquency (age 15)	3406	1.08	1.64	0	13

**Table 2:** Correlations among main study variables

	1	2	3	4	5	6	7	8
1. Prior paternal incarceration (age 1)								
2. Behavior problems (age 5)	.10**							
3. Father engagement (age 5)	− .13**	− .14**						
4. Paternal current Incarceration (age 5)	.38**	.09**	− .16**					
5. Behavior problems (age 9)	.08**	.54**	− .14**	.08**				
6. Father engagement (age 9)	− .19**	− .19**	.54**	− .24**	− .17**			
7. Paternal current Incarceration (age 9)	.21**	.11**	-.08*	.41**	.09**	− .30**		
8. Teen delinquency (age 15)	.08**	.16**	-.06	.09**	.20**	− .14**	.10**	

^*^p < .01, **p < .001

Missing data analyses indicated that participants not retained by year 15 did not differ on paternal incarceration, paternal education, paternal race, family income-to-poverty ratio, infant temperament, early child behavior problems, child sex, or levels of father engagement. CFA confirmed the model fit of the latent variables, child behavior problems, and father engagement at both year 5 and year 9, *CFI* = 0.951, *RMSEA* = 0.043, CI_90%_[0.041; 0.045]). Standardized factor loadings are shown in supplemental materials.

### Structural Equation Model (SEM): Hypothesized Effects

The main SEM model indicated good model fit, *CFI* = 0.935, *RMSEA* = 0.033, CI_90%_[0.032; 0.034]). Standardized parameter results are presented in Fig. 1. Although small effects, paternal history of incarceration prior to age 1 was associated with higher behavior problems and lower father engagement at age 5. Paternal incarceration prior to age 1 was not related to child behavior problems or level of father engagement at age 9. Father’s current incarceration at age 5 was associated with subsequent father engagement, but not child behavior problems. Child problem behaviors at age 5 were not associated with father engagement at age 9. Father engagement at age 5 was also not associated with subsequent child behavior problems. Paternal incarceration prior to age 1 was not associated with teen self-reported delinquency at age 15, nor was current incarceration at year 5 or year 9. Higher self-reported delinquency at age 15 was uniquely related to more early child behavior problems at age 9. However, father engagement at child ages 5 and 9 and child problem behaviors at age 5 were not related to teen self-reported delinquency. No significant indirect pathways emerged, indicating a lack of support for mediation of an effect of history of incarceration on teen delinquency through either child behavior problems, *β* = 0.01, *p* = 0.024, CI99%[0.00; 0.01], or father engagement, *β* = 0.00, *p* = 0.052, CI99%[0.00; 0.01].

### Structural Equation Model (SEM): Effects of Covariates

Fathers with less than a high school education had children with more behavior problems and fathers with a college degree had children with less behavior problems at age 5 and are more engaged with their children than fathers with a high school degree alone. Black fathers had lower reported levels of engagement with their children at age 5 compared to white fathers. Income-to-poverty ratio was not related to child behavior problems at age 5. Infant temperament was also associated with greater child problem behaviors at age 5.

Fathers with a college degree were more involved at child age 9 than fathers with only a high school degree and black fathers were less engaged at age 9 than white fathers. Hispanic fathers also had children with more behavior problems at age 9, compared to white fathers. Child temperament and income-to-poverty ratio at age 1 were not associated with father engagement or child behavior problems at age 9. Youth with black fathers had higher reported teen delinquency compared to youth with white fathers. Paternal education, child temperament, and family income-to-poverty ratio were not associated with teen delinquency.

### Multigroup Moderation

To test moderation by child sex, we used an omnibus test in which the freely estimated model was compared to a fully constrained model. These results showed that the fully constrained model did not fit significantly worse than the freely estimated multigroup model, Δ*χ*^2^(14) = 27.541, *p* = 0.016, indicating that the paths from paternal history of incarceration to later teen delinquency were not moderated by child sex. Paths for the freely estimated multigroup model are shown in supplemental materials.

## Discussion

Paternal incarceration is an important risk factor for subsequent teen delinquency and can have lasting effects on children and adolescents (Porter & King, 2015; Turney, 2017a). However, little research has focused on potential mediators throughout development. The current study extended the literature using a longitudinal design to consider the long-term role of paternal incarceration prior to early childhood, as well as the concurrent associations with incarceration during childhood. We utilized a four-wave design over the course of a 15-year period to examine whether early child behavior problems and father engagement mediate the effect of paternal history of incarceration on delinquency in middle adolescence. The findings of the current study provide a nuanced understanding of these relationships.

Consistent with the previous literature, results indicate that paternal incarceration is associated with the emergence of early behavior problems in children, and subsequently, early behavior problems escalate into teen delinquency. Specifically, paternal incarceration prior to the child’s first year was linked to increased early child behavior problems at age 5. However, paternal history of incarceration did not have a lasting association with child behavior problems by age 9. This suggests that the immediate effects of paternal incarceration on child behaviors might attenuate over time. Furthermore, the temporal gap between paternal history of incarceration prior to early childhood and the teen years might diminish the direct impact. Interestingly, paternal incarceration during ages 5 and 9 was not related to subsequent externalizing behavior problems. Over time, other factors and experiences may be of greater importance to the child’s development, such as peer relationships, school experiences, and neighborhood factors. These influences may become more salient as children age and act as protective factors.

Incarceration has long-term adverse consequences for fathers including greater social and economic hardships, as well as reduced accessibility to their children (Geller, 2013; Woldoff & Washington, 2008). However, longitudinal investigation of the relationship between paternal incarceration and subsequent levels of engagement has been lacking. Paternal incarceration has been associated with lower levels of engagement (Dwyer Emory, 2018), and in turn, low levels of father engagement have been linked to children’s problematic behaviors (Tautolo et al., 2015). Results of the current study support the adverse association between paternal incarceration, both history of and concurrent incarceration, and subsequent levels of father engagement in early childhood. However, contrary to expectations, father engagement was not related to subsequent engagement in delinquent behavior in the current study. Future studies should continue to consider the complex nature of father engagement and its relationship to child outcomes.

Contrary to expectations, the overall model revealed no sex differences in the links among paternal incarceration, early childhood behavior problems, father engagement, and teen delinquency. Previous literature has been mixed, with some studies indicating that boys may be more influenced by their father’s incarceration than girls (Foster & Hagan, 2013), whereas others did not evidence sex differences (Murray & Farrington, 2005). The lack of findings in the current study may indicate that other contextual factors may be more influential in the relationship. Future research should continue to consider the complex nature of the relationships between these variables and the potential impacts on both boys and girls.

### Implications

Overall, the findings highlight the importance of addressing the long-term consequences associated with paternal incarceration by highlighting some of the possible mechanisms involved in that relationship. In order to minimize the influence of paternal incarceration on families, increasing parenting programs that target and support positive relationships between incarcerated men and their families may be beneficial, specifically by targeting a father’s engagement with his family and children. Past research has demonstrated that these types of family-based interventions have merit (Welsh & Farrington, 2015). The findings in the current study also highlight the importance of early detection of and interventions addressing behavior problems in children, as they may lead to more successful efforts to prevent teen delinquency (Murray, 2010; Shaw et al., 2006). Findings from this study also contribute to discussions of criminal justice policy reform by emphasizing the broader familial impacts of incarceration, beyond just the incarcerated individual. Given the systematic barriers, such as issues attaining housing and employment (Phelps, 2018), in life after prison, programs targeted at reducing resource strain may be particularly important for fathers in order to mitigate some of the negative associations of parental incarceration. Furthermore, findings bring attention to the importance of considering the potential compounding influence of paternal incarceration history on child outcomes.

### Strengths, Limitations, and Future Directions

The present study had several strengths, which enabled the examination of the mediation hypotheses over critical periods of development using robust methodology. A key strength was the use of a longitudinal design spanning a 15-year period. The study design allowed for the examination of the relationship between paternal incarceration and the emergence of teen delinquency over the course of adolescence, as well as the modeling of temporal separation between the predictor, both mediators, and the outcome.

One of the major limitations of the current study revolves around the measure of father engagement, as the measure only encompassed a small number of activities. A more robust measure of father engagement should be included in future research in order to advance our understanding of the role that fathers play in their child’s development. Another limitation lies with the measurement of paternal history of incarceration, as we were unable to ascertain when the fathers were incarcerated, and if they remained incarcerated for later timepoints. As prior incarceration is a risk factor for future incarceration (Carson & Golinelli, 2012) and it is possible that some fathers remained incarcerated for the duration of the data collection, future studies should consider measuring changes in incarceration history and the length of the incarceration, as reincarceration may also play a role in level of father engagement.

Other risk factors that may play a mediating role, such as peer (Slagt et al., 2015) and neighborhood influences (Graif, 2015), which may account for the relationship between paternal incarceration and subsequent teen delinquency should be considered. Future studies should continue to consider the interplay of individual, social, and contextual factors that may contribute to the emergence of delinquent behaviors. Intervention studies should also be utilized to experimentally test mediation in the context of efforts to prevent the development of teen delinquency.

## Supplementary Information

Below is the link to the electronic supplementary material.

Supplementary file1 (DOCX 39 KB)

## Data Availability

FFCWS data are publicly available at https://ffcws.princeton.edu/

## References

[CR1] Achenbach, T. M., & Rescorla, L. A. (2000). *Manual for the ASEBA preschool forms and profiles* (Vol. 30). University of Vermont, Research center for children, youth, & families.

[CR2] Assink, M., van der Put, C. E., Hoeve, M., de Vries, S. L. A., Stams, G. J. J. M., & Oort, F. J. (2015). Risk factors for persistent delinquent behavior among juveniles: A meta-analytic review. *Clinical Psychology Review,**42*, 47–61. 10.1016/j.cpr.2015.08.002 26301752 10.1016/j.cpr.2015.08.002

[CR3] Basto-Pereira, M., Começanha, R., Ribeiro, S., & Maia, Â. (2015). Long-term predictors of crime desistance in juvenile delinquents: A systematic review of longitudinal studies. *Aggression and Violent Behavior,**25*, 332–342. 10.1016/j.avb.2015.09.012

[CR4] Besemer, S., Ahmad, S. I., Hinshaw, S. P., & Farrington, D. P. (2017). A systematic review and meta-analysis of the intergenerational transmission of criminal behavior. *Aggression and Violent Behavior,**37*, 161–178. 10.1016/j.avb.2017.10.004

[CR5] Bright, C. L., Sacco, P., Kolivoski, K. M., Stapleton, L. M., Jun, H.-J., & Morris-Compton, D. (2017). Gender differences in patterns of substance use and delinquency: A latent transition analysis. *Journal of Child & Adolescent Substance Abuse,**26*(2), 162–173. 10.1080/1067828X.2016.1242100 28603406 10.1080/1067828X.2016.1242100 PMC5461976

[CR6] Burke, J. D., Pardini, D. A., & Loeber, R. (2008). Reciprocal relationships between parenting behavior and disruptive psychopathology from childhood through adolescence. *Journal of Abnormal Child Psychology,**36*(5), 679–692. 10.1007/s10802-008-9219-7 18286366 10.1007/s10802-008-9219-7 PMC2976977

[CR7] Buss, A. H., & Plomin, R. (1984). Theory and measurement of EAS. *Temperament: Early Developing Personality Traits,**84*, 104.

[CR8] Carson, E. A., & Golinelli, D. (2012). *Prisoners in 2012: advance counts*. US Department of Justice, Office of Justice Programs, Bureau of Justice Statistics.

[CR9] Carter, A. (2019). The consequences of adolescent delinquent behavior for adult employment outcomes. *Journal of Youth and Adolescence,**48*(1), 17–29. 10.1007/s10964-018-0934-2 30298224 10.1007/s10964-018-0934-2

[CR10] Davison, K. K., Charles, J. N., Khandpur, N., & Nelson, T. J. (2017). Fathers’ perceived reasons for their underrepresentation in child health research and strategies to increase their involvement. *Maternal and Child Health Journal,**21*(2), 267–274. 10.1007/s10995-016-2157-z 27473093 10.1007/s10995-016-2157-z PMC5500207

[CR11] Dwyer Emory, A. (2018). Explaining the consequences of paternal incarceration for children’s behavioral problems. *Family Relations,**67*(2), 302–319. 10.1111/fare.12301

[CR12] Farrington, D. P. (2011). Families and crime. In J. Wilson & J. Petersilla (Eds.), *Crime and public policy* (pp. 130–157). New York: Oxford University Press.

[CR13] Flouri, E., Midouhas, E., & Narayanan, M. K. (2016). The relationship between father involvement and child problem behaviour in intact families: A 7-year cross-lagged study. *Journal of Abnormal Child Psychology,**44*(5), 1011–1021. 10.1007/s10802-015-0077-9 26349744 10.1007/s10802-015-0077-9

[CR14] Foster, H., & Hagan, J. (2013). Maternal and paternal imprisonment in the stress process. *Social Science Research,**42*(3), 650–669. 10.1016/j.ssresearch.2013.01.008 23521986 10.1016/j.ssresearch.2013.01.008

[CR15] Gargano, L. M., Locke, S., Li, J., & Farfel, M. R. (2018). Behavior problems in adolescence and subsequent mental health in early adulthood: Results from the World Trade Center Health Registry Cohort. *Pediatric Research,**84*, 205–209. 10.1038/s41390-018-0050-8 29907850 10.1038/s41390-018-0050-8 PMC6185774

[CR16] Geller, A. (2013). Paternal incarceration and father–child contact in fragile families. *Journal of Marriage and Family,**75*(5), 1288–1303. 10.1111/jomf.12056 24839304 10.1111/jomf.12056 PMC4022283

[CR17] Geller, A., Garfinkel, I., Cooper, C. E., & Mincy, R. B. (2009). Parental incarceration and child well-being: Implications for urban families*. *Social Science Quarterly,**90*(5), 1186–1202. 10.1111/j.1540-6237.2009.00653.x 20228880 10.1111/j.1540-6237.2009.00653.x PMC2835345

[CR18] Geller, A., Garfinkel, I., & Western, B. (2011). Paternal incarceration and support for children in fragile families. *Demography,**48*, 25–47. 10.1007/s13524-010-0009-9 21318455 10.1007/s13524-010-0009-9 PMC3220952

[CR19] Goncy, E. A., & van Dulmen, M. H. M. (2010). Fathers do make a difference: Parental involvement and adolescent alcohol use. *Fathering: A Journal of Theory, Research, and Practice about Men as Fathers,**8*(1), 93–108. 10.3149/fth.0801.93

[CR20] Graif, C. (2015). Delinquency and gender moderation in the moving to opportunity intervention: The role of extended neighborhoods. *Criminology,**53*(3), 366–398. 10.1111/1745-9125.12078

[CR21] Hoeve, M., Dubas, J. S., Eichelsheim, V. I., van der Laan, P. H., Smeenk, W., & Gerris, J. R. M. (2009). The relationship between parenting and delinquency: A meta-analysis. *Journal of Abnormal Child Psychology,**37*(6), 749–775. 10.1007/s10802-009-9310-8 19263213 10.1007/s10802-009-9310-8 PMC2708328

[CR22] Hu, L., & Bentler, P. M. (1999). Cutoff criteria for fit indexes in covariance structure analysis: Conventional criteria versus new alternatives. *Structural Equation Modeling: A Multidisciplinary Journal,**6*(1), 1–55. 10.1080/10705519909540118

[CR23] King, V., & Sobolewski, J. M. (2006). Nonresident fathers’ contributions to adolescent well-being. *Journal of Marriage and Family,**68*(3), 537–557. 10.1111/j.1741-3737.2006.00274.x 18270550 10.1111/j.1741-3737.2006.00274.x PMC2239255

[CR24] Lamb, M. E. (2000). The history of research on father involvement. *Marriage & Family Review,**29*(2–3), 23–42. 10.1300/J002v29n02_03

[CR25] Lamb, M. E. (2010). *The role of the father in child development*. (5^th^ Ed.). John Wiley & Sons.

[CR26] Lerner, R. M., & Kauffman, M. B. (1985). The concept of development in contextualism. *Developmental Review,**5*(4), 309–333. 10.1016/0273-2297(85)90016-4

[CR27] Leve, L. D., & Cicchetti, D. (2016). Longitudinal transactional models of development and psychopathology. *Development and Psychopathology,**28*(3), 621–622. 10.1017/S0954579416000201 27427795 10.1017/S0954579416000201

[CR28] Little, T. D., Cunningham, W. A., Shahar, G., & Widaman, K. F. (2002). To parcel or not to parcel: Exploring the question, weighing the merits. *Structural Equation Modeling: A Multidisciplinary Journal,**9*(2), 151–173. 10.1207/S15328007SEM0902_1

[CR29] Lundberg, S., McLanahan, S., & Rose, E. (2007). Child gender and father involvement in fragile families. *Demography,**44*(1), 79–92. 10.1353/dem.2007.0007 17461337 10.1353/dem.2007.0007

[CR30] Maruschak, L. M., Bronson, J., & Alper, M. (2021). *Parents in prison and their minor children: survey of prison inmates, 2016*. Bureau of Justice Statistics*. *Retrieved April 21, 2023 fromhttps://www.ojp.gov/library/publications/parents-prison-and-their-minor-children-survey-prison-inmates-2016

[CR31] Mathiesen, K. S., & Tambs, K. (1999). The EAS Temperament Questionnaire—factor structure, age trends, reliability, and stability in a Norwegian sample. *Journal of Child Psychology and Psychiatry,**40*(3), 431–439. 10.1111/1469-7610.00460 10190344

[CR32] Mayes, S. D., Castagna, P. J., & Waschbusch, D. A. (2020). Sex differences in externalizing and internalizing symptoms in ADHD, autism, and general population samples. *Journal of Psychopathology and Behavioral Assessment,**42*(3), 519–526. 10.1007/s10862-020-09798-4

[CR33] Murray, D. W. (2010). Treatment of preschoolers with attention-deficit/hyperactivity disorder. *Current Psychiatry Reports,**12*(5), 374–381. 10.1007/s11920-010-0142-6 20676944 10.1007/s11920-010-0142-6

[CR34] Murray, J., & Farrington, D. P. (2005). Parental imprisonment: Effects on boys’ antisocial behaviour and delinquency through the life-course. *Journal of Child Psychology and Psychiatry,**46*(12), 1269–1278. 10.1111/j.1469-7610.2005.01433.x 16313427 10.1111/j.1469-7610.2005.01433.x

[CR35] Murray, J., & Farrington, D. P. (2008). The effects of parental imprisonment on children. *Crime and Justice,**37*, 133–206. 10.1086/520070

[CR36] Murray, J., Janson, C.-G., & Farrington, D. P. (2007). Crime in adult offspring of prisoners: A cross-national comparison of two longitudinal samples. *Criminal Justice and Behavior,**34*(1), 133–149. 10.1177/0093854806289549

[CR37] O’Hara, M. W., & Fisher, S. D. (2010). Psychopathological states in the father and their impact on parenting. In S. Tyano, M. Keren, H. Hermann, & J. Cox (Eds.), *Parenthood and mental health: A bridge between infant and adult psychiatry* (pp. 231–240). Chichester, UK: Wiley. 10.1002/9780470660683.ch21

[CR38] Ogg, J., & Anthony, C. J. (2019). Parent involvement and children’s externalizing behavior: Exploring longitudinal bidirectional effects across gender. *Journal of School Psychology,**73*, 21–40. 10.1016/j.jsp.2019.02.002 30961879 10.1016/j.jsp.2019.02.002

[CR39] Paz, Y., Orlitsky, T., Roth-Hanania, R., Zahn-Waxler, C., & Davidov, M. (2021). Predicting externalizing behavior in toddlerhood from early individual differences in empathy. *Journal of Child Psychology and Psychiatry,**62*(1), 66–74. 10.1111/jcpp.13247 32645218 10.1111/jcpp.13247

[CR40] Pettit, B., & Lyons, C. J. (2009). Incarceration and the legitimate labor market: Examining age-graded effects on employment and wages. *Law & Society Review,**43*(4), 725–756. 10.1111/j.1540-5893.2009.00387.x

[CR41] Phelps, M. S. (2018). Ending mass probation: Sentencing, supervision, and revocation. *The Future of Children,**28*(1), 125–146. 10.1353/foc.2018.0006

[CR42] Poehlmann-Tynan, J., & Turney, K. (2021). A developmental perspective on children with incarcerated parents. *Child Development Perspectives,**15*(1), 3–11. 10.1111/cdep.12392

[CR43] Porter, L. C., & King, R. D. (2015). Absent fathers or absent variables? A new look at paternal incarceration and delinquency. *Journal of Research in Crime and Delinquency,**52*(3), 414–443. 10.1177/0022427814552080

[CR44] Roettger, M. E., & Swisher, R. R. (2011). Associations of fathers’ history of incarceration with sons’ delinquency and arrest among Black, White, and Hispanic males in the United States*. *Criminology,**49*(4), 1109–1147. 10.1111/j.1745-9125.2011.00253.x

[CR45] Sampson, R. J., & Loeffler, C. (2010). Punishment’s place: The local concentration of mass incarceration. *Daedalus,**139*(3), 20–31. 10.1162/DAED_a_00020 21032947 10.1162/daed_a_00020 PMC3043762

[CR46] Sarkadi, A., Kristiansson, R., Oberklaid, F., & Bremberg, S. (2008). Fathers’ involvement and children’s developmental outcomes: A systematic review of longitudinal studies. *Acta Paediatrica,**97*(2), 153–158. 10.1111/j.1651-2227.2007.00572.x 18052995 10.1111/j.1651-2227.2007.00572.x

[CR47] Schwartz-Soicher, O., Geller, A., & Garfinkel, I. (2011). The effect of paternal incarceration on material hardship. *Social Service Review,**85*(3), 447–473. 10.1086/661925 24839314 10.1086/661925 PMC4020140

[CR48] Shaw, D. S., Dishion, T. J., Supplee, L., Gardner, F., & Arnds, K. (2006). Randomized trial of a family-centered approach to the prevention of early conduct problems: 2-year effects of the family check-up in early childhood. *Journal of Consulting and Clinical Psychology,**74*(1), 1–9. 10.1037/0022-006X.74.1.1 16551138 10.1037/0022-006X.74.1.1

[CR49] Slagt, M., Dubas, J. S., Deković, M., Haselager, G. J. T., & van Aken, M. A. G. (2015). Longitudinal associations between delinquent behaviour of friends and delinquent behaviour of adolescents: Moderation by adolescent personality traits. *European Journal of Personality,**29*(4), 468–477. 10.1002/per.2001

[CR50] Steffensmeier, D., & Schwartz, J. (2009). Trends in girls’ delinquency and the gender gap: statistical assessment of diverse sources. In *The Delinquent Girl* (pp. 50–83). Temple University Press. Retrieved September 4, 2023 from http://www.scopus.com/inward/record.url?scp=71049138270&partnerID=8YFLogxK

[CR51] Swisher, R. R., & Waller, M. R. (2008). Confining fatherhood: Incarceration and paternal involvement among nonresident White, African American, and Latino fathers. *Journal of Family Issues,**29*(8), 1067–1088. 10.1177/0192513X08316273

[CR52] Sykes, B. L., & Pettit, B. (2015). Severe deprivation and system inclusion among children of incarcerated parents in the United States after the Great Recession. *RSF: The Russell Sage Foundation Journal of the Social Sciences,**1*(2), 108–132.

[CR53] Tautolo, E.-S., Schluter, P. J., & Paterson, J. (2015). Pacific father involvement and early child behaviour outcomes: Findings from the Pacific Islands Families Study. *Journal of Child and Family Studies,**24*(12), 3497–3505. 10.1007/s10826-015-0151-5

[CR54] Thompson, R., Tabone, J. K., Litrownik, A. J., Briggs, E. C., Hussey, J. M., English, D. J., & Dubowitz, H. (2011). Early adolescent risk behavior outcomes of childhood externalizing behavioral trajectories. *The Journal of Early Adolescence,**31*(2), 234–257. 10.1177/0272431609361203

[CR55] Turney, K. (2015). Liminal men: Incarceration and relationship dissolution. *Social Problems,**62*(4), 499–528. 10.1093/socpro/spv015

[CR56] Turney, K. (2017a). The unequal consequences of mass incarceration for children. *Demography,**54*(1), 361–389. 10.1007/s13524-0 28063011 10.1007/s13524-016-0543-1

[CR57] Turney, K. (2017b). Unmet health care needs among children exposed to parental incarceration. *Maternal and Child Health Journal,**21*(5), 1194–1202. 10.1007/s10995-016-2219-2 28108834 10.1007/s10995-016-2219-2

[CR58] Uggen, C., & McElrath, S. (2014). Parental incarceration: What we know and where we need to go. *Journal of Criminal Law and Criminology,**104*, 597.

[CR59] Welsh, B. C., & Farrington, D. P. (2015). Monetary value of early developmental crime prevention and its policy significance commentary. *Criminology & Public Policy,**14*(4), 673–680.

[CR60] Wilbur, M. B., Marani, J. E., Appugliese, D., Woods, R., Siegel, J. A., Cabral, H. J., & Frank, D. A. (2007). Socioemotional effects of fathers’ incarceration on low-income, urban, school-aged children. *Pediatrics,**120*(3), e678–e685. 10.1542/peds.2006-2166 17766508 10.1542/peds.2006-2166 PMC2423929

[CR61] Wildeman, C. (2010). Paternal incarceration and children’s physically aggressive behaviors: Evidence from the Fragile Families and Child Wellbeing Study. *Social Forces - SOC FORCES,**89*, 285–309. 10.2307/40927563

[CR62] Woldoff, R. A., & Washington, H. M. (2008). Arrested contact: The criminal justice system, race, and father engagement. *The Prison Journal,**88*(2), 179–206. 10.1177/0032885508319154

[CR63] Wothke, W. (2000). *Longitudinal and multigroup modeling with missing data*. Psychology Press.

[CR64] Yoder, J. R., Brisson, D., & Lopez, A. (2016). Moving beyond fatherhood involvement: The association between father–child relationship quality and youth delinquency trajectories. *Family Relations,**65*(3), 462–476. 10.1111/fare.12197

