# Supplementary material for: Paternal Incarceration and Adolescent Delinquency: Role of Father Engagement and Early Child Behavior Problems
Source: Prev Sci. 2024 Oct 2;25(8):1228–37. doi: 10.1007/s11121-024-01734-2 (PMC11652568; doi:10.1007/s11121-024-01734-2)
Supplement: Supplementary file 1 — Supplementary file1 (DOCX 39 KB) [file 11121_2024_1734_MOESM1_ESM.docx]

**Paternal Incarceration and Adolescent Delinquency: Role of Father Engagement and Early Child Behavior Problems**

Prevention Science

Abigail J Anderson, Christopher C Henrich, and Sylvie Mrug

University of Alabama at Birmingham

**Author Note**

Abigail J Anderson
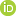
 https://orcid.org/0009-0008-9391-7761

Christopher C Henrich
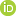
 [https://orcid.org/ 0000-0002-4957-0325](about:blank)

Sylvie Mrug
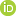
 https://orcid.org/[0000-0001-5426-8713](https://orcid.org/0000-0001-5426-8713)

*CFA with standardized indicators*

| **Latent Variable** | **Parcels** | **λ** | ***SE*** | **𝑝** |
| --- | --- | --- | --- | --- |
| Child | Aggressive Behaviors | .69 | .02 | <.001 |
| Behavior | Destructive Behaviors | .63 | .02 | <.001 |
| Problems | Disruptive Behaviors | .70 | .01 | <.001 |
| (age 5) | Vocal Behaviors | .69 | .01 | <.001 |
|  | Temperament | .65 | .02 | <.001 |
|  | Delinquent Behaviors | .67 | .01 | <.001 |

| **Latent Variable** | **Parcels** | **λ** | ***SE*** | **𝑝** |
| --- | --- | --- | --- | --- |
| Child | Aggressive Behaviors | .77 | .02 | <.001 |
| Behavior | Destructive Behaviors | .72 | .02 | <.001 |
| Problems | Disruptive Behaviors | .78 | .01 | <.001 |
| (age 9) | Vocal Behaviors | .79 | .01 | <.001 |
|  | Temperament | .66 | .02 | <.001 |
|  | Delinquent Behaviors | .69 | .02 | <.001 |

| **Latent Variable** | **Parcels** | **λ** | ***SE*** | **𝑝** |
| --- | --- | --- | --- | --- |
| Father | Peak-a-boo and Sing to | .83 | .01 | <.001 |
| Engagement | Read and Tell Stories | .90 | .01 | <.001 |
| (age 5) | Play together inside and visit relatives | .80 | .01 | <.001 |
|  | Show Affection and Put child to bed | .71 | .01 | <.001 |

| **Latent Variable** | **Parcels** | **λ** | ***SE*** | **𝑝** |
| --- | --- | --- | --- | --- |
| Father | Household Chores Together and Play Outside | .83 | .01 | <.001 |
| Engagement | Watch TV and Play Videogames | .78 | .01 | <.001 |
| (age 9) | Indoor Activities Together and Read | .80 | .01 | <.001 |
|  | Talked about Current Events and Day | .86 | .01 | <.001 |
|  | Did Homework Together | .77 | .01 | <.001 |

|  | Child behavior problems (age 5) | | | Father engagement  (age 5) | | | Father incarceration  (age 5) | | | Child behavior problems (age 9) | | | Father engagement  (age 9) | | | Father incarceration  (age 9) | | | Teen delinquency (age 15) | | |
| --- | --- | --- | --- | --- | --- | --- | --- | --- | --- | --- | --- | --- | --- | --- | --- | --- | --- | --- | --- | --- | --- |
|  | **β** | ***SE*** | **𝑝** | **β** | ***SE*** | **𝑝** | **β** | ***SE*** | **𝑝** | **β** | ***SE*** | **𝑝** | **β** | ***SE*** | **𝑝** | **β** | ***SE*** | **𝑝** | **β** | ***SE*** | **𝑝** |
| Paternal incarceration (age 1) | .06 | .02 | .008 | -.10 | .03 | .001 | .35 | .03 | <.001 | .00 | .02 | .903 | -.05 | .03 | .098 | .05 | .03 | .064 | .02 | .02 | .447 |
| Paternal education (age 1) |  |  |  |  |  |  |  |  |  |  |  |  |  |  |  |  |  |  |  |  |  |
| Less than high school | .08 | .03 | .006 | -.00 | .03 | .917 | .07 | .03 | .009 | .02 | .03 | .444 | -.05 | .03 | .046 | ,04 | .03 | .132 | -.01 | .03 | .672 |
| Some college | -.03 | .03 | .282 | .05 | .03 | .077 | -.03 | .02 | .172 | -.00 | .02 | .806 | .04 | .03 | .104 | -.01 | .02 | .687 | -.04 | .02 | .076 |
| College | -.10 | .02 | <.001 | .10 | .02 | <.001 | -.03 | .01 | .026 | -.03 | .02 | .117 | .07 | .02 | .001 | -.02 | .02 | .252 | -.06 | .02 | .013 |
| Paternal race (age 1) |  |  |  |  |  |  |  |  |  |  |  |  |  |  |  |  |  |  |  |  |  |
| Black | .00 | .03 | .775 | -.15 | .03 | <.001 | .08 | .02 | <.001 | -.04 | .02 | .068 | -.09 | .03 | .001 | .03 | .02 | .146 | .09 | .03 | <.001 |
| Hispanic | -.05 | .03 | .102 | -.06 | .03 | .036 | -.01 | .02 | .573 | -.11 | .03 | <.001 | -.04 | .03 | .126 | -.04 | .02 | .032 | .03 | .03 | .174 |
| Other | -.00 | .02 | .903 | -.-5 | .02 | .027 | .03 | .02 | .040 | .01 | .03 | .541 | -.013 | .03 | .046 | -.02 | .01 | .047 | .02 | .02 | .267 |
| Poverty ratio (age 1) | -.03 | .02 | .223 | -.02 | .02 | .395 | -.03 | .03 | .119 | -.04 | .02 | .013 | .02 | .02 | .222 | -.03 | .02 | .128 | -.03 | .02 | .042 |
| Child temperament (age 1) | .16 | .02 | <.001 | -.04 | .02 | .042 | .02 | .02 | .331 | -.03 | .02 | .119 | .01 | .02 | .637 | -.01 | .02 | .491 | .00 | .02 | .952 |
| Child behavior problems (age 5) |  |  |  |  |  |  |  |  |  | .53 | .03 | <.001 | -.07 | .03 | .011 |  |  |  | .04 | .03 | .200 |
| Father engagement (age 5) |  |  |  |  |  |  |  |  |  | -.05 | .03 | .050 | .48 | .02 | <.001 |  |  |  | .05 | .03 | .173 |
| Father incarceration (age 5) |  |  |  |  |  |  |  |  |  | .02 | .03 | .512 | -.10 | .03 | .001 | .37 | .03 | <.001 | .03 | .03 | .290 |
| Child behavior problems (age 9) |  |  |  |  |  |  |  |  |  |  |  |  |  |  |  |  |  |  | .15 | .03 | <.001 |
| Father engagement (age 9) |  |  |  |  |  |  |  |  |  |  |  |  |  |  |  |  |  |  | -.09 | .04 | .015 |
| Father incarceration (age 9) |  |  |  |  |  |  |  |  |  |  |  |  |  |  |  |  |  |  | .03 | .03 | .242 |

*Standardized Coefficients from the Main Structural Equation Model*

*Multigroup-Model with all standardized structural paths freely estimated for Males*

|  | Child behavior problems (age 5) | | | Father engagement  (age 5) | | | Father incarceration  (age 5) | | | Child behavior problems (age 9) | | | Father engagement  (age 9) | | | Father incarceration  (age 9) | | | Teen delinquency (age 15) | | |
| --- | --- | --- | --- | --- | --- | --- | --- | --- | --- | --- | --- | --- | --- | --- | --- | --- | --- | --- | --- | --- | --- |
|  | **β** | ***SE*** | **𝑝** | **β** | ***SE*** | **𝑝** | **β** | ***SE*** | **𝑝** | **β** | ***SE*** | **𝑝** | **β** | ***SE*** | **𝑝** | **β** | ***SE*** | **𝑝** | **β** | ***SE*** | **𝑝** |
| Paternal incarceration (age 1) | .06 | .02 | .008 | -.10 | .03 | .001 | .35 | .03 | <.001 | .00 | .02 | .903 | -.05 | .03 | .098 | .05 | .03 | .064 | .02 | .02 | .447 |
| Paternal education (age 1) |  |  |  |  |  |  |  |  |  |  |  |  |  |  |  |  |  |  |  |  |  |
| Less than high school | .08 | .03 | .006 | -.00 | .03 | .917 | .07 | .03 | .009 | .02 | .03 | .444 | -.05 | .03 | .046 | ,04 | .03 | .132 | -.01 | .03 | .672 |
| Some college | -.03 | .03 | .282 | .05 | .03 | .077 | -.03 | .02 | .172 | -.00 | .02 | .806 | .04 | .03 | .104 | -.01 | .02 | .687 | -.04 | .02 | .076 |
| College | -.10 | .02 | <.001 | .10 | .02 | <.001 | -.03 | .01 | .026 | -.03 | .02 | .117 | .07 | .02 | .001 | -.02 | .02 | .252 | -.06 | .02 | .013 |
| Paternal race (age 1) |  |  |  |  |  |  |  |  |  |  |  |  |  |  |  |  |  |  |  |  |  |
| Black | .00 | .03 | .775 | -.15 | .03 | <.001 | .08 | .02 | <.001 | -.04 | .02 | .068 | -.09 | .03 | .001 | .03 | .02 | .146 | .09 | .03 | <.001 |
| Hispanic | -.05 | .03 | .102 | -.06 | .03 | .036 | -.01 | .02 | .573 | -.11 | .03 | <.001 | -.04 | .03 | .126 | -.04 | .02 | .032 | .03 | .03 | .174 |
| Other | -.00 | .02 | .903 | -.-5 | .02 | .027 | .03 | .02 | .040 | .01 | .03 | .541 | -.013 | .03 | .046 | -.02 | .01 | .047 | .02 | .02 | .267 |
| Poverty ratio (age 1) | -.03 | .02 | .223 | -.02 | .02 | .395 | -.03 | .03 | .119 | -.04 | .02 | .013 | .02 | .02 | .222 | -.03 | .02 | .128 | -.03 | .02 | .042 |
| Child temperament (age 1) | .16 | .02 | <.001 | -.04 | .02 | .042 | .02 | .02 | .331 | -.03 | .02 | .119 | .01 | .02 | .637 | -.01 | .02 | .491 | .00 | .02 | .952 |
| Child behavior problems (age 5) |  |  |  |  |  |  |  |  |  | .53 | .03 | <.001 | -.07 | .03 | .011 |  |  |  | .04 | .03 | .200 |
| Father engagement (age 5) |  |  |  |  |  |  |  |  |  | -.05 | .03 | .050 | .48 | .02 | <.001 |  |  |  | .05 | .03 | .173 |
| Father incarceration (age 5) |  |  |  |  |  |  |  |  |  | .02 | .03 | .512 | -.10 | .03 | .001 | .37 | .03 | <.001 | .03 | .03 | .290 |
| Child behavior problems (age 9) |  |  |  |  |  |  |  |  |  |  |  |  |  |  |  |  |  |  | .15 | .03 | <.001 |
| Father engagement (age 9) |  |  |  |  |  |  |  |  |  |  |  |  |  |  |  |  |  |  | -.09 | .04 | .015 |
| Father incarceration (age 9) |  |  |  |  |  |  |  |  |  |  |  |  |  |  |  |  |  |  | .03 | .03 | .242 |

|  | Child behavior problems (age 5) | | | Father engagement  (age 5) | | | Father incarceration  (age 5) | | | Child behavior problems (age 9) | | | Father engagement  (age 9) | | | Father incarceration  (age 9) | | | Teen delinquency (age 15) | | |
| --- | --- | --- | --- | --- | --- | --- | --- | --- | --- | --- | --- | --- | --- | --- | --- | --- | --- | --- | --- | --- | --- |
|  | **β** | ***SE*** | **𝑝** | **β** | ***SE*** | **𝑝** | **β** | ***SE*** | **𝑝** | **β** | ***SE*** | **𝑝** | **β** | ***SE*** | **𝑝** | **β** | ***SE*** | **𝑝** | **β** | ***SE*** | **𝑝** |
| Paternal incarceration (age 1) | .06 | .02 | .008 | -.10 | .03 | .001 | .35 | .03 | <.001 | .00 | .02 | .903 | -.05 | .03 | .098 | .05 | .03 | .064 | .02 | .02 | .447 |
| Paternal education (age 1) |  |  |  |  |  |  |  |  |  |  |  |  |  |  |  |  |  |  |  |  |  |
| Less than high school | .08 | .03 | .006 | -.00 | .03 | .917 | .07 | .03 | .009 | .02 | .03 | .444 | -.05 | .03 | .046 | ,04 | .03 | .132 | -.01 | .03 | .672 |
| Some college | -.03 | .03 | .282 | .05 | .03 | .077 | -.03 | .02 | .172 | -.00 | .02 | .806 | .04 | .03 | .104 | -.01 | .02 | .687 | -.04 | .02 | .076 |
| College | -.10 | .02 | <.001 | .10 | .02 | <.001 | -.03 | .01 | .026 | -.03 | .02 | .117 | .07 | .02 | .001 | -.02 | .02 | .252 | -.06 | .02 | .013 |
| Paternal race (age 1) |  |  |  |  |  |  |  |  |  |  |  |  |  |  |  |  |  |  |  |  |  |
| Black | .00 | .03 | .775 | -.15 | .03 | <.001 | .08 | .02 | <.001 | -.04 | .02 | .068 | -.09 | .03 | .001 | .03 | .02 | .146 | .09 | .03 | <.001 |
| Hispanic | -.05 | .03 | .102 | -.06 | .03 | .036 | -.01 | .02 | .573 | -.11 | .03 | <.001 | -.04 | .03 | .126 | -.04 | .02 | .032 | .03 | .03 | .174 |
| Other | -.00 | .02 | .903 | -.-5 | .02 | .027 | .03 | .02 | .040 | .01 | .03 | .541 | -.013 | .03 | .046 | -.02 | .01 | .047 | .02 | .02 | .267 |
| Poverty ratio (age 1) | -.03 | .02 | .223 | -.02 | .02 | .395 | -.03 | .03 | .119 | -.04 | .02 | .013 | .02 | .02 | .222 | -.03 | .02 | .128 | -.03 | .02 | .042 |
| Child temperament (age 1) | .16 | .02 | <.001 | -.04 | .02 | .042 | .02 | .02 | .331 | -.03 | .02 | .119 | .01 | .02 | .637 | -.01 | .02 | .491 | .00 | .02 | .952 |
| Child behavior problems (age 5) |  |  |  |  |  |  |  |  |  | .53 | .03 | <.001 | -.07 | .03 | .011 |  |  |  | .04 | .03 | .200 |
| Father engagement (age 5) |  |  |  |  |  |  |  |  |  | -.05 | .03 | .050 | .48 | .02 | <.001 |  |  |  | .05 | .03 | .173 |
| Father incarceration (age 5) |  |  |  |  |  |  |  |  |  | .02 | .03 | .512 | -.10 | .03 | .001 | .37 | .03 | <.001 | .03 | .03 | .290 |
| Child behavior problems (age 9) |  |  |  |  |  |  |  |  |  |  |  |  |  |  |  |  |  |  | .15 | .03 | <.001 |
| Father engagement (age 9) |  |  |  |  |  |  |  |  |  |  |  |  |  |  |  |  |  |  | -.09 | .04 | .015 |
| Father incarceration (age 9) |  |  |  |  |  |  |  |  |  |  |  |  |  |  |  |  |  |  | .03 | .03 | .242 |

*Multigroup-Model with all standardized structural paths freely estimated for Females*
